# Phytochemical Screening, Antifungal, and Anticancer Activities of Medicinal Plants *Thymelaea Hirsuta*, *Urginea Maritima*, and *Plantago Albicans*

**DOI:** 10.1155/2022/9544915

**Published:** 2022-12-30

**Authors:** Esraa Ahmed Mohamed El-Bondkly, Basim Al Shammari, Mervat Morsy Abbas Ahmed El-Gendy, Ibrahim A. Alsafari, Alaa Ahmed Mohamed Ahmed El-Bondkly, Fareed Shawky El-Shenawy, Ahmed Mohamed El-Bondkly

**Affiliations:** ^1^Faculty of Medicine, October 6 University, 6th of October City, Giza, Egypt; ^2^Department of Clinical Laboratory Sciences, College of Applied Medical Science, University of Hafar Al Batin, P. O. Box 1803, Hafar Al Batin 31991, Saudi Arabia; ^3^Chemistry of Natural and Microbial Products Department, National Research Centre, Dokki, Giza 12622, Egypt; ^4^Biology Department, College of Sciences, University of Hafar Al Batin, P. O. Box 1803, Hafar Al Batin 31991, Saudi Arabia; ^5^Faculty of Pharmacy, Cairo University, Kasr El-Aini, Cairo 11562, Egypt; ^6^Botany and Microbiology Department, Faculty of Science, Al-Azhar University, Asyut Branch, Asyut, Egypt; ^7^Genetics and Cytology Department, National Research Centre, Dokki, Giza 12622, Egypt

## Abstract

Ethyl acetate, ethanol, and acetone extracts of the medicinal plants *Thymelaea hirsuta* L., *Urginea maritima* L., and *Plantago albicans* L. (aerial parts) were evaluated for their phytochemical compositions, antimycotic activity against dermatophytes, and antiproliferative activity against different human cancer cell lines. Among them, the ethanolic extracts showed the highest phytochemical contents along with hyperactivities and were then selected for gas chromatography-mass spectrometry and Fourier-transform infrared spectroscopy analysis. The Fourier-transform infrared spectroscopy analysis confirmed the presence of different characteristic peak values with various functional chemical groups of the active components. However, *U. maritima* extracts through Fourier-transform infrared spectroscopy analysis showed distinctive peaks related to phenolic, amines, amides, aromatic, alkanes, alkyne, cyclopentanone, conjugated aldehyde, nitro, methoxy, uronic acids, aromatic esters, tertiary alcohol or ester, secondary and primary alcohols, aliphatic ether, sulfoxide, vinylidene, and halo compounds. Many bioactive main compounds with reported biological activities were detected by GC/MS (%) in the ethanolic extract of *T. hirsuta*, *U. maritima*, and *P. albicans*. All studied dermatophytes included a diverse set of virulence factors, including phospholipase, protease, keratinase, hemolysis, and melanoid production, all of which play vital roles in dermatophytic infection. Ethanolic extract of *P. albicans* inhibited the growth of *Trichophyton* soudanense totally and *Trichophyton erinacei* in addition to all *Microsporum* species. In contrast, the ethanolic extract of *Trichophyton hirsuta* at concentrations of 25 g/mL totally prevented the growth of all *Trichophyton* species. EtOH extract of *U. maritima* completely prevented the growth (100% inhibition) of all dermatophytic strains under study at the lowest concentration of 12.5 *μ*g/mL. Scanning electron microscope analysis revealed considerable morphological modifications and structural alterations in dermatophyte species exposed to ethanolic extract of these plants. The viability of HCT-116, HepG-2, MCF-7, and HeLa cell lines was reduced after treatment with the ethanolic extracts of *T. hirsuta*, *U. maritima*, and *P. albicans* individually with IC50 values (10.0, 9.97, 48.5, and 56.24 *μ*g/mL), (26.98, 25.0, 17.11, and 9.52 *μ*g/mL), and (9.32, 7.46, 12.50, and 16.32 *μ*g/mL), respectively. Our work revealed the significance of these traditional ethnomedical plants as potent sources for biologically active pharmaceuticals with potential applicability for the treatment of fungal and cancer diseases.

## 1. Introduction

Regardless of advances in oncology, cancer remains one of the leading causes of morbidity and mortality worldwide [1, 2]. In 2020, about 19.3 million new cancer cases were recorded worldwide, resulting in almost 10 million deaths. Furthermore, it may spread throughout the body and numerous organs through metastasis, which includes migration and invasion [3]. Recently, there have been many traditional treatments for cancer control, including chemotherapy, surgery, radiotherapy, and immunotherapy. Still, unfortunately, there are some important limitations to their use due to lack of safety, efficiency, undesired side effects, high cost, and availability [4]. As a result, natural products with anticancer qualities have received a lot of attention since they are safe, low-cost, and widely accessible. In addition, they contain many biological and therapeutic activities that may promote health and relieve cancer [1, 5].

On the other hand, dermatophytes are keratinophilic fungi, including *Microsporum*, *Epidermophyton*, and *Trichophyton* genera, which penetrate exclusively into the tissues of the stratum corneum, hair or nails using nonenzymatic (hemolysin and melanoid formation) and proteases, keratinases, cellulases, and phospholipases are enzymatic virulence agents that cause infection [6, 7]. Dermatophyte infections in humans, particularly those affecting the skin and mucosal surface, are a severe concern, particularly in tropical and subtropical areas, where they are the most common pathogen. [8]. Dermatophytic fungal infections are a real public health problem, especially among patients with weakened immune systems (lymphomas, human immunodeficiency virus, cancer, diabetes, and transplants). Especially due to the modulation of the clinical spectrum of pathogens and the overuse of conventional antifungal drugs, strains are emerging that are resistant to current antifungals, resulting in the search for alternative natural products such as organic extracts from medicinal plants [9].

Aromatic and medicinal plants used for a long time in traditional medicine have been the subject of numerous studies to analyze their chemical composition and evaluate their pharmaceutical properties as antifungal agents for the treatment of various fungal illnesses [10]. El-Demerdash [11] reported that due to Egypt's ecological diversity, the traditional pharmacopoeia contains a vast resource of medicinal plants that have long been used in folk medicine to treat many diseases as demonstrated on temple walls and papyrus, such as the well-known *Ebers Papyrus* of 1550 BC documented 876 medicaments with 328 diverse components derived from many plant species including *Thymelaea hirsuta* L., *Urginea maritima* L., and *Plantago albicans* L. Medicinal plants *T. hirsuta* L., *U. maritima* L., and *P. albicans* L. endemic to Egypt and the coastal Mediterranean region and commonly used as antiulcer, anti-inflammatory, antigiardiasic, antimalarial, anticancer, wound healing, immunomodulatory, antibacterial, antioxidant, hypoglycaemic, hepatoprotective, cytotoxic, and hypolipidemic agents attributed to their high glycosides, flavonoids, reducing compounds, tannins, anthraquinones, mucilage, anthocyanins, fatty acids, polysaccharides, steroids, and terpenes contents [12–17].

Solvent extraction is a process that aims to extract certain active components or secondary metabolites like flavonoids, alkaloids, terpenes, steroids, glycosides, and saponins present in natural resource materials. Using a suitable solvent and standard extraction procedure through the solid/liquid separation operation and then the components of interest are then solubilized and contained within the solvent [1, 3, 6, 18–22]. Yahaya et al. [23] reported that among different plant extracts, the ethanolic extracts of seven medicinal plants showed the highest antifungal properties against different human pathogenic fungi. Among them, the ethanolic extract of leaves of *Boswellia dalzielii*, *Ocimum gratissimum*, and *Crescentia alata* caused potent repression in the growth of *Trichophyton rubrum*, *Trichophyton mentagrophyte*, and *Candida albicans* with the least MIC value (312.5 *μ*g/mL).

The effects of potential bioactive treatments on the ultrastructure and morphology of dermatophytes can be assessed by scanning electron microscopy with customized protocols like the osmium tetroxide (OsO_4_) protocol. It should be preferred over other methods for its unparalleled image quality, resolution, magnification, actual sample structure preservation, and minimal sample loss [24]. As previously reported by Taha et al. [5], morphological changes in hyphae of 30 clinical dermatophyte isolates, including *Trichophyton* and *Microsporum* species, following exposure to ethanolic and acetone extracts of henna leaves such as collapsing, distortion, twisting, inflating, bulging, and crushing of hyphae with corrugation of walls. Depressions on hyphal surfaces and distortion of hyphae at 25 to 75 *μ*g/mL of ethanol or acetone extract were observed using scanning electron microscopy (SEM) analysis. Gas chromatography-mass spectrometry (GC-MS) and Fourier-transform infrared spectroscopy (FTIR) are powerful analytical techniques used to identify and quantify bioactive compounds and functional groups in extracts derived from natural sources [1, 3, 22]. Combining the properties of mass spectrometry and gas chromatography to identify distinct components within the examined sample, GC-MS has shown to be a suitable technology for identifying volatile oils, various hydrocarbons, acids, alkaloids, esters, fatty acids, nitro, and amino compounds [25, 26].

This work is aimed at investigating the biochemical properties of ethyl acetate, acetone, and ethanol extracts of aerial parts of medicinal plants, *T. hirsuta*, *U. maritima*, and *P. albicans*, individually followed by evaluating their potential inhibition activity against a wide range of dermatophytes, including the species of *Trichophyton*, *Microsporum*, and *Epidermophyton* genera. In addition, using HepG-2 and MCF-7 cell lines provide an inexpensive source of pharmacological agents that can protect humans from lethal cancer diseases and invasive dermatophytes against various types of cancers such as liver, colon, breast, and cervix cancers. Likewise, HCT-116 cell lines can provide an inexpensive pharmacological agent source that protects humans from lethal cancer diseases and invasive dermatophytes. Moreover, phytoactive compounds and functional groups present in the ethanolic extracts of these plants' aerial parts were assessed based on IR and GC-MS analysis.

## 2. Materials and Methods

### 2.1. Chemicals

Merck (Darmstadt, Germany) and Sigma-Aldrich Chemical Co. supplied all chemicals (St. Louis, MO).

### 2.2. Chemistry Part

#### 2.2.1. Plant Samples and Preparation of Extracts

**Figure 1:**
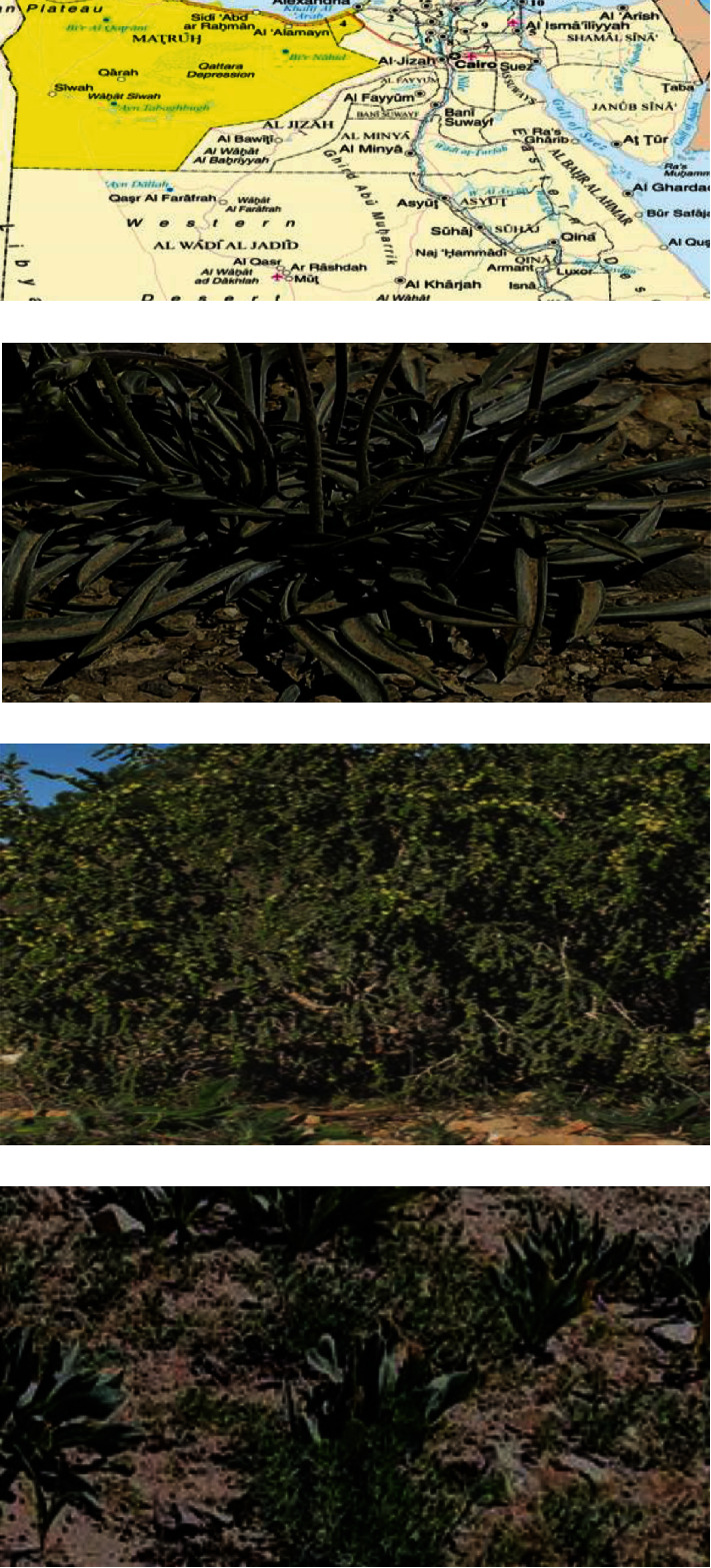
Sidi Brani, Matrouh Governorate, Egypt (a), the medicinal plants *P*. *albicans* (b), *T*. *hirsuta* (c), and *U*. *maritima* (d).

Healthy and fresh and aerial parts of *T. hirsuta*, *U. maritima*, and *P. albicans* were collected from Sidi Brani, Matrouh Governorate, Egypt (N 31°36′39^″^, E 25°55′32^″^) (Figures 1(a)–1(d)) during April-May 2018. The Department of Botany, Faculty of Science, Al-Azhar University, confirmed the plant identification. Each plant was carefully cleaned with tap water, then deionized water, before being air-dried at room temperature in the shade and crushed to a fine powder. Each plant's dried powdered aerial parts were divided into three portions (250 g each). The first was extracted with ethyl acetate, the second with ethanol, and the third with acetone (1 : 10 *w*/*v*), individually on a horizontal shaker at 150 rpm for 72 h at 30°C. The resulting solutions of ethyl acetate, ethanol, and acetone samples were filtered using Whatman No. 1 filter paper in a Büchner funnel. Then, it evaporated under a vacuum in a rotary evaporator. These extracts were stored in a refrigerator at 4°C until further phytochemical and biological analysis. Crude extracts were subjected to preliminary phytochemical analysis, antidermatophytes, and anticancer activity, while only crude ethanolic extracts were subjected to IR and GC–MS analysis.

#### 2.2.2. Preliminary Phytochemical Analysis of the Three Extracts of Each Plant

Phytochemical profiles of the three extracts (ethyl acetate, acetone, and ethanol) for each of the *P. albicans*, *T. hirsuta*, and *U*. *maritima* were evaluated individually. Medoua et al. [27] determined the total polyphenol content by measuring the absorbance at 725 nm in gallic acid equivalents. The modified vanillin HCl technique was used to quantify condensed tannins [28, 29]. As a result of the analysis, milligram catechin equivalents (mg CE/g DW) were calculated based on the absorbance measured at 500 nm. Chang et al. [30] described the aluminum chloride colorimetric technique for measuring total flavonoids. At 415 nm, the absorbance was measured and represented as mg of quercetin equivalents (QE)/g of dry extracts. The carotene reference absorbance was measured at 450 nm using the Kurilich and Juvik [31] method. A gravimetric technique was used to determine the total saponin content [32], while alkaloid content was determined using the Biradar and Rachetti [33] method. Every sample was examined in triplicate.

#### 2.2.3. Fourier Transform Infrared Spectroscopy of Ethanolic Extracts of *U*. *maritima*, *T*. *hirsuta*, and *P*. *albicans*

The Fourier transform infrared spectrophotometer (FTIR) is one of the most effective tools for determining the kinds of chemical bonds (functional groups) present in compounds. To generate transparent sample discs for FTIR analysis, 10 mg of dried ethanolic extract powder from several plants was encapsulated in 100 mg of KBr pellet (FTIR grade). The sample of each plant was loaded in FTIR spectroscope (Broker Vertex80v, Germany), individually with a scan range from 400 to 4000 cm-1 with a resolution of 4 cm-1 to detect the corresponding functional groups that might be involved in the bioactivities. By interpreting the infrared absorption spectrum, the chemical bonds in a molecule can be determined. Spectral differences are the objective reflection of componential differences [34].

#### 2.2.4. Gas Chromatography-Mass Spectrometry (GC-MS) Analysis of the Ethanolic Extract of *U. maritima*, *T. hirsuta*, and *P. albicans* Analysis

GC-MS is an analytical technique that combines the characteristics of gas chromatography and mass spectrometry to identify various compounds in a test sample [26]. The ethanol extracts of *U. maritima*, *T. hirsuta*, and *P. albicans* aerial parts were subjected to GC-MS analysis to identify some potent volatile and semivolatile components. Chemical analysis in each ethanolic plant extract after dissolved in chloroform was analyzed by gas chromatography-mass spectrometry (Thermo Scientific, Trace GC Ultra/ISQ Single Quadrupole MS) and performed in MS1 scan mode using DB-5MS fused-silica column (30 m × 250 *μ*m, 0.25 *μ*m film thicknesses, Agilent) with helium as carrier gas at a constant flow rate of 1 mL/min. Mass spectra were taken at an electron ionization energy of 70 eV. The temperature program (oven temperature) in the gas chromatography was 40°C elevated to 230°C at 5°C/min, and the injection volume was 1 *μ*L. All quantifications were performed using a built-in data-handling application supplied by the gas chromatograph manufacturer. The composition was expressed as a percentage of the overall peak area measured from the base to the peak's tip. The components in each extract were identified by comparing the observed mass spectrum to known spectra from the database of chemicals in the GC-MS NIST and Wiley mass spectra libraries (software, D.03.00) and those in the literature [35–37].

### 2.3. Dermatophytes Part

#### 2.3.1. Isolation and Characterization of Clinical Dermatophytes

Clinical samples were sent to the Mycology laboratory (Faculty of Science, Al-Azhar University) and Chemistry of Natural and Microbial Products Department (National Research Centre) for microscopic examination, cultivation, and identification of dermatophytes based on their colony shape and microscopic examination. The validity of culture methods using different isolation media, including rapid sporulation medium (RSM), dermatophyte test medium (DTM), and dermatophyte identification medium (DIM) for isolation and characterization of dermatophytes was compared as previously described [38–42]. Furthermore, the production of nonenzymatic and enzymatic virulence factors was defined according to Achterman and White [43], Elavarashi et al. [44], and Walailak [45].

#### 2.3.2. Inoculum Preparation and Agar Diffusion Method for Antidermatophytes Assay of Each Plant Extract

Dermatophytic strains were grown in Sabouraud dextrose broth (SDB) for 10 days at 28°C to retain the largest number of conidia. Conidia were collected for analysis following centrifugation at 4°C, suspension in sterile distilled water, and adjusting to 10^5^ conidia/mL. Each fungal solution (100 *μ*L) was separately dispersed over the SDA medium. For determining biological activities, stock solutions from extracts were individually made in dimethyl sulfoxide (DMSO) (DMSO was not exceeding 0.5% in the experiment). To assess the antimycotic activity of each plant extract against each dermatophytic fungus by the disc diffusion method, 10 *μ*L of each extract prepared in DMSO at various concentrations of 25, 50, and 100 g/mL were applied to discs of Whatman paper (No. 1) and placed onto an SDA medium inoculated with one of the tested fungi. Fungal toxicity was reported as a percentage of mycelial growth inhibition and was calculated using the Pandey et al. [46] method.

(1) $$\begin{array}{c} \text{FT}=\frac{\left(\text{dc}-\text{dt}\right)}{\text{dc}}\times 100, \end{array}$$

where FT is the fungal toxicity, dt is the average diameter of the treated fungal colony, and dc is the average diameter of the control fungal colony. As negative and positive controls, agar plates containing 1% DMSO and 100 g/mL clotrimazole were utilized.

#### 2.3.3. Assessment of the Fungal Morphology and Surface Structure Alterations after Treatment with Plant Extracts Using Scanning Electron Microscopy (SEM) Analysis

Each fungal strain was seeded in SDA, incubated for 5 days at 37°C, and inoculated into a tube containing 5 mL of SDB with 1% glucose. Then 500 *μ*L of the inoculated broth (10^6^ CFU/mL) was added in 24-well plates containing glass slides and 500 *μ*L of tested extract at different concentrations of 5.0, 10.0, and 20.0 *μ*g/mL (each treatment, *n = 3* wells). Sterile water (*n = 3* wells) and chloramphenicol (*n* = 3 wells), individually for 5 days at 37°C, were applied as negative and positive controls, respectively. The control and treated fungi were centrifuged for 5 min at 3000 rpm, supernatants were removed, and samples were washed three times with buffered saline. Samples for SEM examination were prepared following Iwasawa et al. [47] and Mio et al. [48]. These samples were fixed with a solution composed of glutaraldehyde 4%, formaldehyde 2.5% in pH 7.2, 0.1 M sodium cacodylate buffer, and overnight at 4°C. The fixed mycelia were washed three times with 0.1 M sodium cacodylate buffer (pH 7.2), then they were postfixed in 2% osmium tetroxide solution for 2 h in the dark. The postfixed mycelia were then rewashed 3 times with 0.1 M sodium cacodylate buffer for 10 min, then dehydrated in grading concentrations of ethanol (25%–90%) at room temperature for 10 min at each concentration, and then dehydrated in 100% ethanol three times for 15 minutes. Furthermore, the dehydrated specimens were dried for 30 minutes with liquid carbon dioxide in a critical point drier (CPD 030, BAL-TEC, Germany). A sputter coater was used to coat the dried samples with gold (SCD 005, BAL-TEC, Germany). Finally, fungal morphology and cell surface before and after each treatment were visualized and photographed at the Central Laboratory, National Research Centre, Egypt, using a scanning electron microscope (SEM Quanta FEG 250 with field emission gun, FEI Company, Netherlands).

### 2.4. Cytotoxicity Part

#### 2.4.1. *In Vitro* Antiproliferative Activity Assay

Antiproliferative activity of plant extracts at various concentrations of 12.5, 25, 50, 75, and 100 *μ*g/mL against various human carcinoma cell lines, including hepatocellular carcinoma (HepG-2), human colon cancer (HCT-116), breast adenocarcinoma (MCF-7), and cervical carcinoma (HeLa) (purchased from American Type Culture Collection; ATCC, and maintained in the proper conditions) was examined, and quantification of cell proliferation was assessed following the 3-(4,5-dimethylthiazol-2-yl)-2,5-diphenyltetrazolium bromide (MTT) assay as described earlier [49]. HepG-2, HCT-116, MCF-7, and HeLa carcinoma cells were seeded at a density of 1 × 104 cells/well on a 96-well flat-bottom microtiter plate and allowed to adhere for 24 hours at 37°C in a CO_2_ incubator. The culture media was then changed with the new medium, and cells were treated at 37°C for 24 hours in a CO_2_ incubator with varying doses of the tested extract of the desired plant. The culture media was then changed with a new medium. Then, 10 *μ*L of MTT solution (5 mg/mL in phosphate buffer solution) was applied to each well. In a CO_2_ incubator, the plate was incubated for 4 hours at 37°C. Next, the medium was aspirated, and the produced formazan crystals were solubilized in a CO_2_ incubator with 50 *μ*L DMSO/well for 30 minutes at 37°C. The intensity of the dissolved formazan crystals (purple hue) was measured at 540 nm using an ELISA plate reader. The activity of cells in the presence of the test solvent (DMSO) is regarded as a control. MTT tests were then run in triplicate for each sample type. The cellular viability is expressed as (sample − blank)/(control − blank) × 100%.

## 3. Results

### 3.1. Chemistry Part

#### 3.1.1. Phytochemical Analysis of Selected Plant Extracts

In line with our results, the content of flavonoids, polyphenols, alkaloids, phytochemicals, and other tannins varied considerably according to the solvents used, solvent polarity, particle size, geographical origin, and extraction methods. As shown in Table 1, high amounts of polyphenols, flavonoids, tannins, carotenoids, lutein, saponins, and alkaloids were detected in the ethanolic extracts of *P. albicans*, *T. hirsuta*, and *U*. *maritima*, respectively (Table 1). Furthermore, our data are consistent with previous studies and found that ethanolic extracts of the aerial parts of *T. hirsuta* gave a high amount of flavonoids, phenol contents, and condensed tannins. The current study recorded the largest amount of phenolic component in all three plant extracts, indicating a wide range of pharmacological and health-promoting effects of these plant extracts (Table 1). Phenolic compounds are essential in diverse nutraceutical, therapeutical, and cosmetic applications. On the other hand, the lowest extractable amounts of these phytochemicals, including polyphenols, flavonoids, tannins, carotenoids, lutein, saponins, and alkaloids, were detected in the ethyl acetate extracts of these plants.

#### 3.1.2. FTIR Spectroscopic Analysis of Aerial Parts Extracts of *P. albicans*, *T. hirsuta*, and *U. maritima*

**Figure 2:**
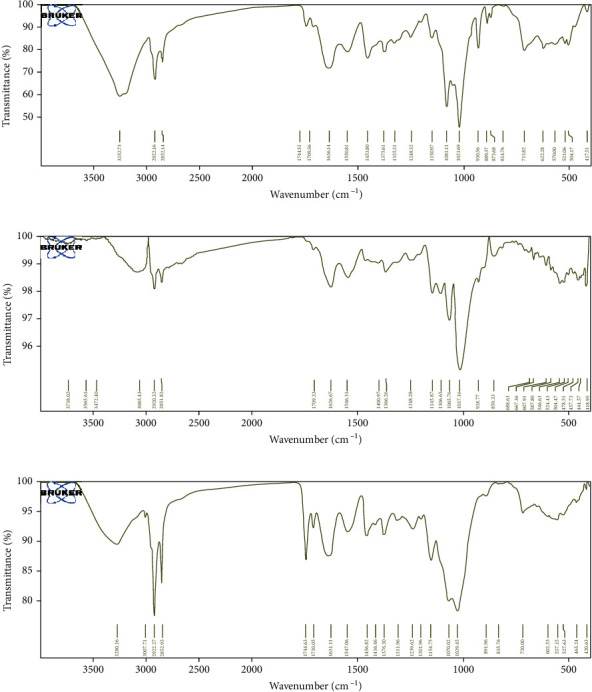
IR spectroscopy of the ethanolic extracts of*. albicans* (a), *T. hirsuta* (b), and *U. maritima* (c).

The current study is aimed at using FTIR spectroscopy to determine the distinctive peak values and functional groups in ethanol extracts of *P. albicans*, *T. hirsuta*, and *U. maritima* aerial parts. The extracts' FTIR analysis showed a variety of distinctive peak values associated with diverse functional components. (Figures 2(a)–2(c)). The characteristic absorption bands in the ethanolic extract of *P*.*albicans* were detected at 3252.73, 2922.16, 2852.14, 1744.51, 1709.36, 1636.14, 1550.81, 1453.80, 1375.61, 1325.21, 1248.32, 1150.97, 1081.11, 1021.69, 930.56, 889.37, 873.68, 814.76, 713.82, 622.28, 570.00, 521.06, 504.17, and 417.51 cm-1 (Figure 2(a)). Among them are the broad characteristic peaks at 3252.73 cm-1 (for O-H stretch, polymeric OH, and hydroxy compound), 2922.16 cm-1 (for hydroxyl, asymmetric stretching of lipids, protein, and carboxylic acids), 2852.14 cm-1 (for C-H stretching and methoxy methyl ether compound), 1709.36 cm-1 (for carbonyl group (C=O) and carbonyl compound), 1636.14 cm-1 (for C=O stretching vibration and ketone group of ketone compounds), 1550.81 cm-1 (for strong N-O stretching nitro compound), 1453.80 cm-1 (for C=C-C, aromatic ring stretch, and aromatic compound), 1375.61, and 1325.21 cm-1 (for O-H bend, alcoholic group, and tertiary alcohol or phenol), 1081.11 cm-1 (for C-O stretch of ether group and cyclic ethers), 1021.69 cm-1 (for phosphate ion and phosphate compounds), 930.56 cm-1 (for P-O-C stretch and aromatic phosphates), 713.82, and 622.28 cm-1 (for C-Br stretch, and aliphatic bromo compounds) were detected (Figure 2(a)). Moreover, the EtOH extract of *T. hirsuta* in Figure 2(b) showed noticeable bands at 3738.02, 3565.61, 3472.40, 3065.43, 2920.32, 2851.82, 1709.33, 1626.67, 1546.31, 1400.97, 1366.26, 1248.28, 1145.87, 1106.65, 1065.76, 1017.16, 928.77, 859.33, 688.63, 667.36, 607.91, 587.80, 546.63, 524.43, 504.47, 478.51, 457.73, and 444.57 cm-1. Among them, the largest peaks were detected at 3065.43 cm-1 (for C-H stretch and aromatic), 2920.32 cm-1 (for asymmetric stretching of –CH (CH2) vibration, lipids, or protein), 1626.67 cm-1 (for C=O stretching vibration, ketone group, and ketone compound), 1546.31 cm-1 (for strong N-O stretching nitro compound), 1366.26 cm-1 (for O-H bend, alcoholic group, and phenol or tertiary alcohol), 1248.28 cm-1 (for CN stretch and primary aromatic amine), 1145.87-1017.16 cm-1 (for PO3 stretch, and phosphate ion), 859.33 cm-1 (for P-O-C stretch and aromatic phosphates), 504.47-444.57 cm-1 (for S-S stretch and aryl disulfides), 546.63-524.43 cm-1 (for strong C-I stretching and iodocompound), and 418.95 cm-1(for aromatic C-OH in-plane bending vibration and phenol). The various functional groups observed in the different extracts probably indicate the presence of carotenoids, carbohydrates, glycogen, amides, calotropin, amino acids, starch, phosphates, calotropogenin, lipids, cellulose, and glycogen. However, ethanolic extract of *U. maritima* through FTIR spectroscopy analysis showed various distinictive absorption peaks at 3280.16 cm-1 (for stretching -OH groups from phenolic compounds or NH/OH stretching of amides and amines), 3007.71 cm-1 (for C-H stretch and aromatic), 2922.27 cm-1 (due to a C-H group asymmetric stretching and hybridized carbon of alkanes), 2852.93 cm-1 (C-H stretch and alkanes), 1744.63 cm-1 (strong C=O stretching and cyclopentanone), 1710.05 cm-1 (strong C=O stretching and conjugated aldehyde), 1631.11 cm-1 (medium C=C stretching, disubstituted (cis), or medium N-H bending, amine), 1547.06 cm-1 (strong N-O stretching nitro compound), 1456.82 cm-1 (medium C-H bending, alkane, and methyl group), 1416.46 cm-1 (could correspond to ester carbonyl groups of the carboxylic function of uronic acids), 1376.30 cm-1 (due to asymmetric in-plane bending of –CH3), 1311.96 cm-1 (O=C-O-C- stretch from the aromatic esters), 1239.62 cm-1 (medium C-N stretching and amine), 1201.96 cm-1 (C-O stretching of tertiary alcohol or ester), 1154.75 cm-1 (strong C-O stretching vibrations from secondary alcohols or aliphatic ether), 1070.02 cm-1 (strong C-O stretching vibrations from primary alcohols), 1029.45 cm-1 (PO3 stretch vibration indicates the presence of phosphate ion or strong S=O stretching of sulfoxide), 891.96 cm-1 (strong C=C bending, alkene, and vinylidene), 835.76 cm-1 (medium C=C bending and alkene), 720.00 cm-1 (bending vibrations of ≡C-H bond of alkyne), 602.53 cm-1 (C-I and iodocompounds), 557.10-527.63 cm-1 (stretching C-Cl or C-I and halo compound), and 465.24-420.63 cm-1 (aromatic C-OH in-plane bending vibration and phenol) (Figure 2(c)).

#### 3.1.3. Gas Chromatography-Mass Spectrometry (GC–MS) Analysis of the Ethanolic Extracts of *P. albicans*, *T. hirsuta*, and *U. maritima*

The main constituents were trans-Caryophyllene (anticancer, antimicrobial, and antioxidant agent; 13.20%); cinnamic acid (antioxidant, antimicrobial, anticancer, neuroprotective, anti-inflammatory, and antidiabetic agent; 12.16%); tetradecane (antioxidant, anticancer, and hypocholesterolemic agent; 11.18%); phytol (anxiolytic, metabolismmodulating, antioxidant, cytotoxic, antinociceptive, apoptosis-inducing, immune-modulating, anti-inflammatory, and antimicrobial agent; 10.21%); catechin (antimicrobial, antiviral, anti-inflammatory, antiallergenic, and anticancer agent; 8.30%); benzofuranone (antitumor, antimicrobial, antioxidative, and antiviral agent; 7.10%); chlorogenic acid (hepatoprotective, antioxidant, cardioprotective, antipyretic, anti-inflammatory, neuroprotective, antiviral, antiobesity, antihypertension, and antimicrobial,; 6.30%); lauric acid (antifungal, antibacterial, and antiviral; 6.26%); naphthalene (anticancer, antimicrobial, and anti-inflammatory agent; 6.12%); arachidic acid (antimicrobial, antifungal, antioxidant, and lipid mediator; 2.26%); glycerin (anti-inflammatory agent; 2.15%); and D-carvone (antibacterial and antisprouting agent; 2.13%) (Table 2). Moreover, lesser amounts (peak area > 2%), including 4-hydroxy-3-methoxybenzoic acid, diathiapentene, 9-eicosene, (E)-octadecanoic acid, methyl ester, hexadecanoic acid, hydroxy-B ionone, pentadecanone, gallic acid, and tetracosanoic acid, were detected in the ethanolic extract of *P. albicans* and also in line with our results, the identification of 22, 19, and 17 chemicals for roots, leaves, and flowers of the medicinal plant *P. decaisnei* by GC-MS analysis, and the prevalent organic compounds were phenolics (55.43%), alkaloids (62.03%), terpenoids (25.59%), fatty acids (42.51%), phytosterols (15.68%), and esters (32.08%); namely, 9, 12, 15-octadecatrien-1-ol (37.45%), roemerine (70.44%), hexadecanoic acid (33.72%), *γ*-sitosterol (11.22%), and decarbomethoxytabersonine (24.49%) with antioxidant and anticancer activities. Furthermore, the phytochemical analysis of the *T. hirsuta* ethanolic extract by GC–MS characterized a total of 25 compounds. The main components in descending order were valencene (antioxidant and cytotoxic agent; 18.0%) > *α*-humulene (antitumor, antioxidant, anti-inflammatory, and antimicrobial agent; 15.25%) > alloaromadendrene (antioxidant, antimicrobial, and antiproliferative agent; 13.60%) > nonadecene (antituberculosis, anticancer, and antioxidant agent; 12.17%) > 3,5-dithiahexanol 5,5-dioxide (antitumour activity; 7.23%) > *σ*-cadinene (antimicrobial, antioxidant, and cholinesterase inhibitor; 4.71%) > lavandulyl acetate (antimicrobial and antiparasitic agent; 4.43%) > *α*-cedrene (antileukemic, antimicrobial, and antiobesity; 3.30%) > *β*-selinene (anti-inflammatory, analgesic, and antipyretic agent; 2.15%) > *β*-bourbonene (antimicrobial and cytotoxicity; 2.12%) > thymohydroquinone dimethyl ether (antibacterial and cytotoxic activities; 2.11%) >1-hexadecanol (antioxidant, antibacterial, and antifungal; 2.00%) > *γ*-gurjunene (free radical scavenging activity; 1.65%) > tetratetracontane (antioxidant and cytoprotective activities; 1.48%) > *α*-guaiene (anti-inflammatory, antinociceptive, and antioxidant; 1.34%) > germacrene-D (antimicrobial and insecticidal; 1.23%) > citronellyl formate (antioxidant, antiinflammatory, and wound healing; 1.19%) > phenol, 2,4-bis (1,1-dimethylethyl)- (antiinflammatory and antibacterial; 1.14%) > *α*-gurjunene (antimicrobial and antioxidant; 1.10%) (Table 3). Conversely, the lowest peak area < 1.0% was recorded for camphor (0.93%), followed by *β*-cyclocitral (0.70%), *α*-pinene (0.61%), tetradecamethyl-heptasiloxane (0.59%), 1-octen-3-ol (0.50%), and phenylethyl alcohol (0.47%)(Table 3). Among the bioactive compounds detected in *U. maritima* ethanolic extract (Table 4), the highest peak areas were detected for 5-isopropyl-2-methyl phenol (antioxidant, antibacterial, antifungal, anticancer, anti-inflammatory, hepatoprotective, spasmolytic, and vasorelaxant agent; 15.21%); eugenol (antibacterial, anticancer, antiviral, and cytotoxic agent; 13.25%); carveol (anticancer; 9.74%); tetrafluorophthalonitrile (fungicidal; 7.62%); hexadecanoic acid (fungicidal, antibacterial, anticancer, and insect attractants; 7.60%); *α*-cadinol (cytotoxic agent; 7.43%); *β*-elemene (anticancer activity in multifarious cancer and antibacterial; 6.58%); 1,2- benzendicarboxylic acid, di-isooctyl ester (fungitoxic and cytotoxic agent; 6.22%); *β*-caryophyllene (antimicrobial, anticancer, cytotoxic, and antioxidant agent; 4.29%); torreyol (cytotoxic, pesticide, and termiticide agent; 4.11%); cyclohexane, 1,4-dimethyl-2-octadecyl- (anticancer; 3.29%); geraniol (antibacterial, antiviral, antitumor, and cytotoxic agent; 2.20%); and borneol (antinociceptive, anti-inflammatory, and cytotoxic agent against tumor cell lines; 1.28%) (Table 4). However, the lower peak areas ≤ 1.0 were detected for linalyl acetate (1.0%); *α*–pinene (0.96%); tetradecanoic acid, methyl ester (0.91%); p-vinylguaiacol (0.83%); cyclohexasiloxane, dodecamethyl (0.76%); oleic acid (0.72%); *β*-myrcene (0.70%); terpinolene (0.68%); 3-carene (0.63%); 2-Pentyl-furan (0.58%); octadecane, 3-ethyl-5-(2-ethylbutyl)- (0.51%); espatulenol (0.45%); *β*-sesquiphellandrene (0.43%); 2-hydroxy-4-methoxybenzaldehyde (0.42%); phenylalcohol (0.35%), and retinol (0.30%).

### 3.2. Dermatophytes Part

#### 3.2.1. Specification and Validation of Dermatophytes Differentiation Media

Fungus culture assists in the identification of certain fungal species. Among the 283 isolates produced following RSM culture, 253 isolates (89.399%) were confirmed dermatophytes (Table 5). According to our findings, many nondermatophytic fungi exhibit a culture morphology similar to dermatophytes. They can affect the color of RSM and DTM, which may be linked to quick growth fungus and resistance to the antifungal antibiotic in DTM, resulting in many false positive findings. These results highlighted the critical need for more affirmative and selective media. Furthermore, our data demonstrated that the DTM medium is not entirely specific for dermatophytes due to owing 18 nondermatophytic fungi (spore-forming bacilli, *S. aureus*, 2, *Escherichia coli*, 2, Candida species, 10) can grow on it, and 94.44% of them can do so. At the same time, 33 true dermatophytic isolates could not do so. The color of the DTM medium changed from yellow to bright red after cultivating because dermatophytes need proteins, resulting in ammonium ion release and an alkaline environment. However, it is not widely utilized because of the high number of false positives generated by quick growers and other pollutants. The findings in Table 5 demonstrated that the DIM medium could remove the false positive outcomes acquired using commercial market media such as RSM and DTM media. After 96 hours of incubation, all dermatophytic isolates (*n* = 253) obtained from RSM could grow on DIM and change color to dark purple. Still, among the 30 nondermatophytes obtained from the RSM medium, only three *Candida* isolates could grow on DIM, and one of them altered its color. Furthermore, owing to the low color intensity and typical creamy bacteria such as *Candida* growth that is clearly distinguished from dermatophytes, the outcome of the positive color change of *Candida* strain on the DIM medium may be disregarded. As a result of these two criteria, the DIM medium specificity and sensitivity for dermatophytic fungus might range from 99.647 to 100.0%. DIM has improved specificity, effectivity, and validity against dermatophytes due to its increased cycloheximide concentration, 4 mg/mL, and incubation at 37°C, as previously described.

#### 3.2.2. Evaluation of the Virulent Factors in Dermatophytes

The highest keratinase productivity was detected in *Microsporum cookie* followed by *Epidermophyton floccosum*, *Trichophyton vaoundei*, and *Trichophyton rubrum* (23.56, 22.08, 20.14, and 20.35 U/mL), respectively but the lowest yields were estimated in *Microsporum racemosum*, *Trichophyton mentagrophytes*, and *Trichophyton soudanense* (9.28, 11.82, and 13.25 U/mL), respectively. Dermatophytic fungal pathogens such as *Trichophyton rubrum*, *Trichophyton mentagrophytes*, *Microsporum gypseum*, and *Microsporum canis* were isolated from the keratin substances with high keratinase productivity. Furthermore, the stratum corneum was the best substrate for lipase production in 46.67% (*M. persicolor*; 62.25, *M. racemosum*; 60.34, *M. ferrogenium* 60.12; *T. erinacei*; 57.38, *T. mentagrophytes*; 54.56, *T. vaoundei*; 36.19, and *T. soudanense*; 29.45 U/mL). Moreover, the nail was the best substrate for lipase production in 33.33% of isolates including *T. tonsurance*, *M. gypseum*, *M. audouinii*, *M. canis*, and *T. rubrum* to produce 40.16, 36.02, 32.01, 23.47, and 23.46 U/mL, respectively) but hair as substrate gave 53.84, 42.88, and 16.49 U/mL of lipase from *E. floccosum*, *M. cookie*, and *Microsporum gallinae*, respectively, (Table 6). Also, in line with our results, all dermatophytic isolates including the species of *Trichophyton*, *Microsporum*, and *E. floccosum* are expressed as keratinase, elastase, protease, phospholipase, and lipase as enzymatic virulence factors along with hemolysin activity which plays a major role in the pathogenesis of infectious diseases. Interestingly, protease productivity in *Microsporum ferrogenium*, *T. rubrum*, *M. canis*, *T. tonsurance*, *M. cookie*, *E. floccosum*, *T. vaoundei*, *M. audouinii*, *T. erinacei*, *M. gallinae*, *T. mentagrophytes*, and *M. racemosum* reached its maximum values with stratum corneum as substrate (Table 6). Furthermore, *T. soudanense* and *M. gypseum* produced maximum protease productivity with hair as substrate, while nail-supported protease formation in *M. persicolor* (17.67 U/mL) (Table 6). On the other hand, *T. soudanense* prefers human hair as a substrate for maximum melanin yield (0.49 mg/mL). Melanoid pigment generation was supported by the human nail as one of the most active nonenzymatic virulence factors by these dermatophytic fungi under research, with productivity ranging from 0.44 to 0.77 mg/mL in all dermatophytic strains (Table 6). The highest amounts of melanoid formation were recorded for *M. gallinae*, *T. rubrum*, *E. floccosum*, *T. erinacei*, *T. tonsurance*, and *M. gypseum* (0.76, 0.77, 0.74, 0.69, 0.71, and 0.68 mg/mL, respectively) on the human nail. Also, that putative targets identified as novel antifungal agents to combat dermatophytes include virulence factors such as melanoid formation, hemolysis, proteases, keratinase, lipase, elastases, sulfate transporters as well as heat shock genes or proteins, and ATP binding cassette. Furthermore, as shown in Table 6, for the hemolysis productivity at *A*_550_, changing the substrates has no significant effect. In general, the highest hemolysin productivity was associated with *T. rubrum*, *T. mentagrophytes*, *E. floccosum*, *M. gypseum*, *M. audouinii*, *M. persicolor*, and *M. ferrogenium* (*A*_550_ = 0.69, 0.68, 0.62, 0.60, 0.53, 0.50, and 0.49, respectively) using stratum corneum as a substrate (Table 6).

#### 3.2.3. Evaluation of the Antifungal Efficacy of Each Plant Extract against Dermatophytes

The ethanolic extract of *P. albicans* could totally inhibit the growth of *T. soudanense* and *T. erinacei* as well as all *Microsporum* species at a concentration of 25 *μ*g/mL. Still, it completely inhibited the growth of *T. tonsurance*, *T. mentagrophytes*, and *T. vaoundei* strain at a higher dose equal to 50 *μ*g/mL (Table 7). Data in Table 4 indicated that among all *Trichophyton* strains, *T. rubrum* showed the highest resistance against the EtOH extract of *P. albicans*, which caused its toxicity by 36.31 ± 0.25, 70.00 ± 0.49, and 85.0 ± 0.65% at a concentration of 12.5, 25.0 and 50 *μ*g/mL, respectively. Moreover, all *P. albicans* extracts exhibited little toxicity on the viability of *E. floccosum* after treatment with EtOAc, EtOH, and acetone extracts of *P. albicans*, respectively, (Table 7). Our results reported that among the aqueous, ethanol, and DMSO extracts of selected medicinal plants *Lantana camara*, *Catharanthus roseus*, *Nerium indicum*, *Ziziphus mauritiana*, and *Sida cordifolia* leaves; ethanol extract of *C. roseus* followed by *N. indicum*, L*. camara*, and *Z. mauritiana* recorded the highest antimycotic activity against fungal pathogens. However, the growth of all *Trichophyton* species and *E. floccosum* was completely inhibited (100% inhibition) by the ethanolic extract of *T. hirsuta* at a concentration of 25 *μ*g/mL and acetone extract at 50 *μ*g/mL (Table 7).

#### 3.2.4. Scanning Electron Microscopy (SEM) Assessment of Morphological Alteration of Exposed and Unexposed Fungi to Plant Extracts at Different Concentrations

**Figure 3:**
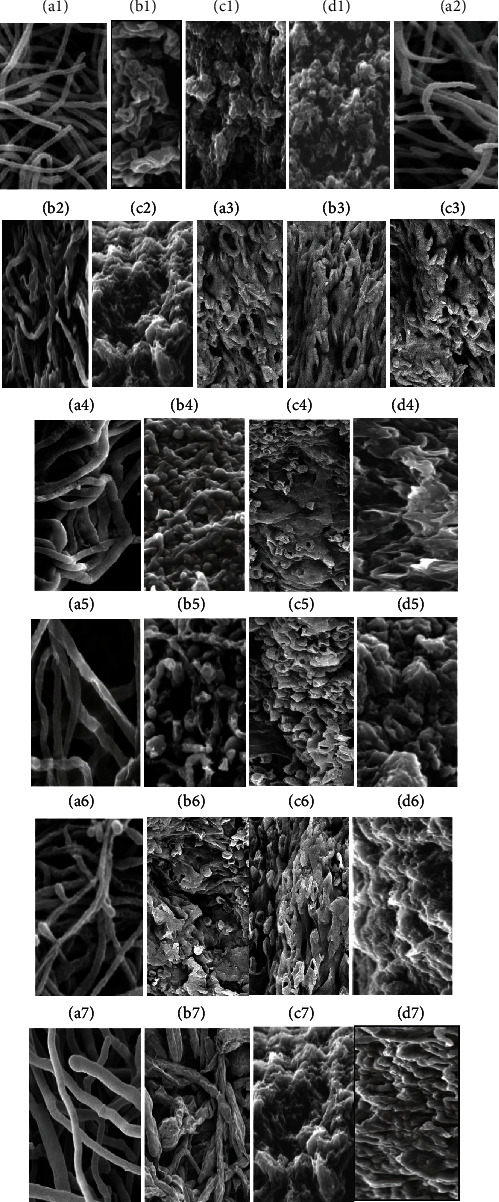
Electron micrographs of the dermatophytes with and without ethanolic plant extracts. *T. rubrum*: control (a1), treated with the extract of *T. hirsuta* (b1–d1), *P. albicans* (a2–c2), and *U. maritima* (a3–c3); *T. mentagrophytes*, untreated (a4), and treated with *U. maritima* (b4–d4); *M. canis*, control (a5) and treated with *T. hirsuta* (b5–d5); *M. gypseum*, untreated (a6) and treated with *T. hirsuta* (b6–d6), and *E. floccosum*, untreated (a7), and treated with the *P. albicans* extract (b7–d7). Scale bar; 10 *μ*m.

Success against dermatophytes requires a deeper understanding of the interactions between the surface of fungal cells and antifungal agents. The SEM analysis provided an interesting method to assess the effect of this plant extract on treated fungal surfaces in contrast to traditional methods that provide a quantitative estimation of antimicrobials based on MICs determinants. The analysis of conducted electron micrographs SEM in Figure 3 revealed the interaction of the *T. rubrum* with extracts of *T. hirsuta* (Figures 3(b1)–3(d1)), *P. albicans* (Figures 3(a2)–3(c2)), and *U. maritima* (Figure 3(a3)–3(c3)). *T. mentagrophytes* treated with *U. maritima* extract (Figures 3(b4)–3(d4)), *M. canis* treated with *T. hirsuta* extract (Figures 3(b5)–3(d5)), *M. gypseum* treated with *T. hirsuta* extract (Figures 3(b6)–3(d6)), *E. floccosum* treated with *P. albicans* extract (Figures 3(b7)–3(d7)) at different concentrations of 5.0, 10.0, and 20.0 *μ*g/mL, respectively. The data in Figure 3 showed that the untreated cells (control) of *T. rubrum*, *T. mentagrophytes*, *M. canis*, *M. gypseum*, and *E. floccosum* (Figures 3(a1), 3(a4), 3(a5), 3(a6), and 3(a7), respectively) were smooth and had regular, thread-like tubular shapes with intact cells morphology. On the other hand, all the plant extracts used could affect the normal structure of the fungal cells and cause severe morphological and cell surface abnormalities that increased with increasing doses of these extracts from 5.0 to 20 *μ*g/mL. Compared to the regular hyphae with no changes in tubular appearance in the control hyphae of *T. rubrum*, the SEM of *T. rubrum* after treatment with EtOH extract of *T. hirsuta* ranged from 5.0 to 20 *μ*g/mL exhibited random irregular clumps structures with the surface demolition, distorted, collapsed, and irreversibly damaged mycelium in addition to the cave formations and trabecula can be observed that seems to be derived from the fusion of hyphae concentrations (Figures 3(b1)–3(d1)). Moreover, *T. rubrum* treated with *P. albicans* extract at a concentration of 5.0, 10.0, and 20.0 *μ*g/mL, respectively (Figures 3(a2)–3(c2)) had a ribbon-like structure with a rough, and fluffy surface, and enlarged hyphal tips with abnormal, and irregular shapes at the lower concentration but at the higher dose of the extract 20 *μ*g/mL, it forms a compact mass with contorted, rough, and distorted hyphae. After treatment with *U. maritima* extract (Figure 3(a3)–3(c3)), large morphological deformations occurred in the *T. rubrum* mycelium shape and hyphae. Hyphae were fused and collapsed forming abnormal and irregular shapes such as wrinkles spongy, conical, and caves structures with rough and granular-like surfaces and bulging, crushing of hyphae with corrugation of walls (Figure 3(a3)–3(c3)). The deformation and fusion increased in the exposed hyphae with increasing the concentration of the extract.

The electron micrographs of control *T. mentagrophytes* hypha without any treatment was presented smoothly to lobed-like surface hyphae, tubular structures, and straight shapes wrapped around each other (Figure 3(a4)). After treatment of *T. mentagrophytes* mycelial with *U. maritima* extract, SEM images showed significant morphological abnormalities in the structural patterns, including swollen, wrinkled, missing turgidity, and slightly cracked hyphae with a rough surface (Figure 3(b4)) at 5.0 *μ*g/mL. Highly compact mycelium with fused hyphae together to form a woven structure that is occasionally interrupted by areas with the appearance of stretch marks in addition to crushed and much-suppressed hyphae, strange formations with the appearance of burst balloons and crush zones were observed at 10 *μ*g/mL (Figure 3(c4)). With increasing the dose of EtOH extract of *U. maritima* to 20 *μ*g/mL, abnormality, irregular, shrinkage, and fusion of hyphae resulted in the cave shape formations of trabecula along with the presence of spongy consistency structures with notable pitting on hyphal walls that indicating a possible loss of the intracellular contents were observed (Figure 3(d4)). These morphological alterations can be suggested to crush the fungal structures of *T. mentagrophytes* caused by *U. maritima* extract. The control hyphae of *M. canis* in Figure 3(a5) revealed smooth walls in normal tubular appearance hyphae. However, *M. canis* hypha treated with ethanolic extract of *T. hirsuta* (5.0, 10.0, and 20.0 *μ*g/mL) in Figures 3(b5), 3(c5), and 3(d5), respectively, had abnormal and irregularly shaped, wrinkled, contorted, and rough macroconidia patterns, rough, twisted, and granular-like surfaces; depressions on hyphal surfaces with a compact mass of contorted, rough, and crushed hyphae. The unexposed mycelium of *M. gypseum* to these fungal extracts showed a typical cigar-shaped morphology, lengthened hyphae of constant diameter with a rounded or lightly tapering apex and a smooth external surface (Figure 3(a6)). Conversely, *M. gypseum* samples treated with *T. hirsuta* extract at a concentration of 5.0, 10.0, and 20.0 *μ*g/mL caused altered mycelium characteristics such as the appearance of knobs on the surface, the abundant presence of abnormal wrinkling, broken down and crushing of hyphal regions with rough and granular-like surface structures and formation of large, irregular, highly wrinkled structures after crushing and fusing of hyphae with corrugation of walls (Figures 3(b6), 3(c6), and 3(d6), respectively). In line with our results, SEM analysis showed that the extract of *Dryopteris fragrans* L. caused mycelial morphology changes like collapsing, twisting, shrinking, and even flattening in *T. rubrum*. SEM micromorphological analysis (Figures 3(b7)–3(d7)) of the *E. floccosum* mycelium treated with the active substances of *P. albicans* extract under study presented notable morphological abnormalities and alterations concerning the controls (Figure 3(a7)). Morphological structure alterations were observed in treated *E. floccosum*. These morphological changes included elongated club-shaped macroconidia that appeared in clusters followed by rupture or bursting of its structures, contracted and swollen tubes, swollen apexes, collapsing, distortion, twisting, inflating, bulging, and crushing of hyphae; depressions on hyphal surfaces, and the mycelium appeared as consistent tangled or folded spongy structures (Figure 3(b7)–3(d7)).

### 3.3. Cytotoxicity Part

#### 3.3.1. Determination of the Antitumor Activity of each Plant Extract against Human Cancer Cell Lines

**Figure 4:**
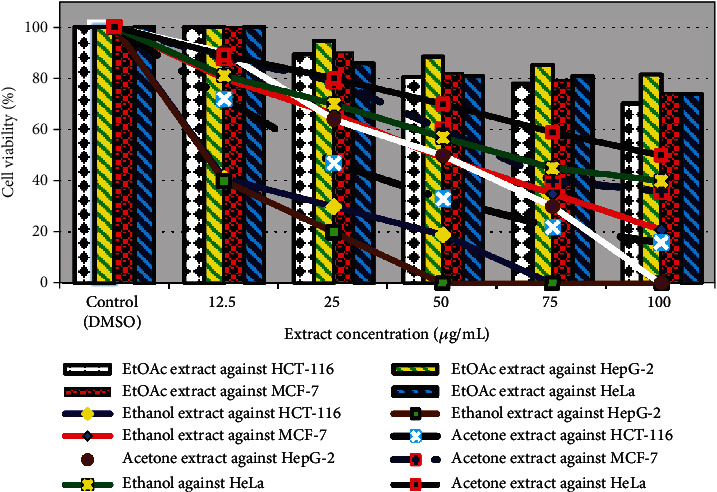
Effect of *P. albicans* extracts against HepG-2, MCF-7, HCT-116, and HeLa carcinoma cell lines.

**Figure 5:**
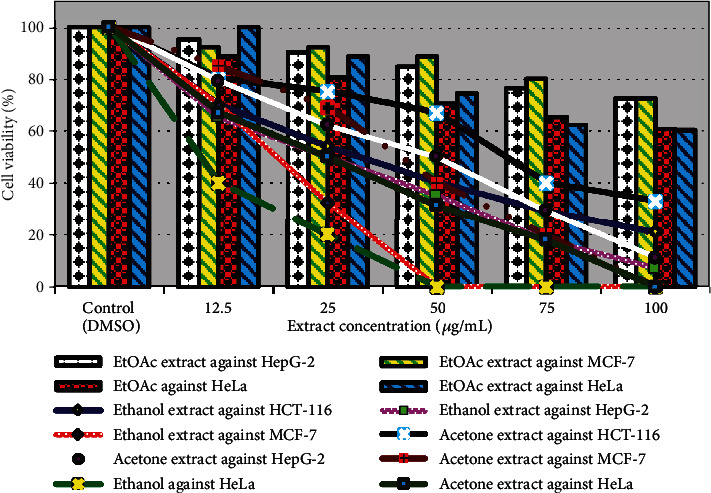
Effect of *T. hirsuta* extracts against HepG-2, MCF-7, HCT-116, and HeLa carcinoma cell lines.

**Figure 6:**
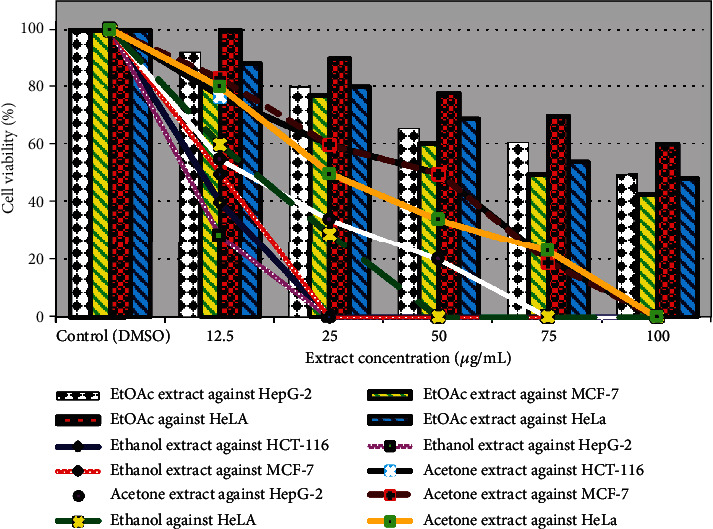
Effect of *U. maritima* extracts against HepG-2, MCF-7, HCT-116, and HeLa carcinoma cell lines.

Nature offers us several chemicals classed as secondary metabolites from plants and has been exploited to discover novel cancer therapies since ancient times. Therefore, this study was started to explore the potential anticancer activity of selected medicinal plants *P. albicans*, *T. hirsuta*, and *U. maritima* aerial parts, as shown in Figures 4–6. The viability of the HCT-116, HepG-2, MCF-7, and HeLa cell lines were reduced to (41%, 30%, 19%, 5%, and 0%), (40%, 20%, 0%, 0%, and 0%), (80%, 66%, 49%, 35%, and 21%), and (81%, 70%, 57%, 45%, and 40%) after treatment with the ethanolic extract of *P. albicans* at a dose of 12.5, 25, 50, 75, and 100 *μ*g/mL, respectively (Figure 4). Moreover, the acetone extract reduced their viability by (72%, 47%, 33%, 22%, and 16%); (90%, 64%, 50%, 30%, and 0%); (88%, 79%, 60%, 40%, and 36%); and (89%, 80%, 70%, 59%, and 50%) at dosage of 12.5, 25, 50, 75, and 100 *μ*g/mL, respectively (Figure 4). The IC50 estimated for the ethanol extract of *P. albicans* was to be 10.0, 9.97, 48.5, and 56.24 *μ*g/mL compared to 23.82, 50.0, 61.52, and 100 *μ*g/mL for the acetone extract against HCT-116, HepG-2, MCF-7, and HeLa cells, respectively (Figure 5). Furthermore, death in HCT-116, HepG-2, MCF-7, and HeLa carcinoma cell lines equal to (30%, 46%, 60%, 71%, and 79%); (35%, 50%, 65%, 82%, and 93%); (30%, 68%, 100%, 100%, and 100%); and (60%, 80%, 100%, 100%, and 100%) was recorded after treatment with ethanol extract of *T. hirsuta* with IC50 values 26.98, 25, 17.11, and 9.52 *μ*g/mL, respectively, (Figure 5). Furthermore, with the acetone extract, the growth of these cell lines was suppressed by (20%, 25%, 33%, 60%, and 67%); (21%, 38%, 50%, 71%, and 89%); (15%, 31%, 60%, 80%, and 100%); and (33%, 50%, 69%, 82%, and 100%) at concentrations of 12.5, 25, 50, 75, and 100 *μ*g/mL, respectively, with IC50; 50.0, 37.42, 60.25, and 25.0 *μ*g/mL against HepG-2, MCF-7, HCT-116, and HeLa cells, respectively (Figure 5). Previously, polar extracts of *T. hirsuta* exhibited a dose-dependent cytotoxic effect on human colon cancer cell growth, and HeLa cell viability with IC50 value of 175 *μ*g/mL. Among all the plants under study, the ethanolic extract of *U. maritima* exhibited the highest antiproliferative against HCT-116, HepG-2, and MCF-7 cell lines (Figure 6). The viability of HepG-2, HCT-116, and MCF-7 cell lines were repressed at 25 *μ*g/mL with IC50; 7.46, 9.32, and 12.50 *μ*g/mL, respectively. Moreover, HeLa cells were completely killed at 50 *μ*g/mL with IC50; 16.32 *μ*g/mL of EtOH - *U. maritima* extract (Figure 6). Furthermore, the acetone extract of *U. maritima* was able to reduce the viability of MCF-7, HCT-116, and HeLa cell lines to zero at a concentration of 100 *μ*g/mL as well as HepG-2 cells at a dose of 75 *μ*g/mL with IC50 equal to 50.0, 50.0, 25.0, and 15.34 *μ*g/mL, respectively (Figure 6).

## 4. Discussion

Nature supplies us with several chemicals known as secondary metabolites that come from plants and have been exploited since ancient times to discover novel remedies. In general, the phytochemical investigation of the *T. hirsuta*, *U. maritima*, and *P. albicans* plants revealed the presence of significant amounts of various chemical components with a history of pharmacological effects, and these quantities increased with the increasing of solvent polarity and reached a peak in ethanol extract of all these plants. In line with our results, Yahyaoui et al. [50–52] stated that the content of flavonoids, polyphenols, tannins, phytochemicals, and other alkaloids varied considerably according to the solvents used, extraction methods, solvent polarity, particle size, and geographical origin. Also, our data agree with Yahaya et al., the present work is aimed at analyzing the ethanol extracts of the aerial parts of *T. hirsuta*, *U. maritima*, and *P. albicans* using the FTIR spectroscopy method for detecting the characteristic peak values and their functional groups. FTIR analysis revealed different characteristic peak values with various functional constituents in the extracts, and our data are in line with Pakkirisamy et al. [53]. Data are consistent with El-Bondkly and El-Gendy [1]; they stated that the FTIR spectrum is the major authoritative technique used for recognizing the chemical functional groups of the active components present in the extract based on peak values in the region of IR radiation. GC-MS is a “gold standard” for medical substances and is one of the most important instruments used to analyze a sample with volatile constituents. Chromatographic analysis of the EtOH extract of *P. albicans* in Table 5 enabled the identification of 21 compounds with different well-known biological activities. The presence of several bioactive compounds detected after GC-MS analysis using the ethanolic extract of *T. hirsuta* justifies the use of leaves for various elements by traditional practitioners. Previously different biological activities were detected for these identified compounds, such as antitumor, antioxidant, anti-inflammatory, and antimicrobial for *α*-humulene; antioxidant, antibacterial, and antiproliferative for alloaromadendrene; antioxidant, and cytotoxicity for valencene; as well as antituberculosis, anticancer, and antioxidant for nonadecene [54]. GC-MS analysis proved that *P. albicans*, *T. hirsuta*, and *U. maritima* plants used in traditional medicine contain a wide range of substances that can be used to treat chronic, cancerous, and infectious diseases. [55–59].

Fungal culture aids in the definitive identification of fungal species. The data in Table 5 indicated that the RSM medium gave low specificity for dermatophytes, then there were many false results due to 45 dermatophytic fungi which were unable to change the RSM medium coloration while 15 nondermatophytes (*Candida* sp.; 11, the spore-forming bacterium of *Bacillus* sp.; 1, *Escherichia coli*; 1, and *Staphylococcus aureus*; 2 isolates) were able to change the RSM medium color. Dermatophytes are keratinophilic fungi closely related to a unique group of organisms, mostly composed of 3 main genera, including *Trichophyton*, *Microsporum*, and *Epidermophyton* that infect keratinous tissue (nails, hairs, and skin) of live human hosts, and other animals to produce infection [60]. In line with our results, Jangid and Begum [61] stated that dermatophytes are fungal pathogens of animals and humans that infect the keratinized tissues, e.g., nails, hairs, and skin, since they produce several kinds of virulence factors for infection, such as keratinase, sulfite, exoprotease, and endoprotease to penetrate into the stratum corneum of the host tissues and produce disease. Shanmuga et al. [62] reported that the hemolytic factor plays a vital role in dermatophytic infections because of the cytotoxic effects on RBC and phagocytic cells, which results in the formation of pores and lysis. According to Elavarashi et al. [44], dermatophyte species secrete several enzymatic virulence activities such as phospholipase, lipase, protease, and gelatinase that hydrolyze host tissue components as a source of nutrients, act as antigens, and stimulate varying degrees of inflammation, as well as nonenzymatic virulence factors such as hemolysin and CAMP-like factors. Yahyaoui et al. [50] reported that medicinal plant; *T. hirsuta* extracts were previously reported to have antimicrobial, antihypoglycemic, antioxidant activities and antitumor due to their high content of phenolic compounds. On the other hand, among all dermatophytes under study, all species of *Microsporum* showed the highest resistant toward the extracts of *T. hirsuta in* descending order *M. racemosum > M. ferrogenium > M. audouinii > M. gallinae > M. persicolor > M. cookie > M. gypseum > M. canis*. Also, Felhi et al. [15] study revealed that the aerial parts extracted from *T. hirsuta* exhibited moderate to strong antimicrobial effects against bacteria and yeasts. While the various constituents of medicinal plants have long been prescribed to treat various infectious diseases, there is little information available regarding their use in fungal skin infections [1, 6, 63]. Among all plants under study, ethyl acetate extracts showed the lowest inhibitory activity against all dermatophytes isolates, but ethanol extracts showed the highest antifungal activity. The polarity of the solvent and the solubility of the antifungal metabolites in ethanol followed by acetone, but not in ethyl acetate, are thus compatible. These results are similar to those of Ghahfarokhi et al. [64], who described hyphal abnormalities such as shrinkage or surface demolition, and enlarged hyphal tips in *T. rubrum* and *T. mentagrophytes* when treated with onion extracts. Massiha and Muradov [65] reported that ethanol extract from different plants, including *Calendula officinalis*, *Acacia arabica*, *Altheae officinalis*, *Ginkgo biloba*, *Juglans regia*, *Hypericum perforatum*, *Solanum nigrum*, *Osimum basilicum*, *Urtica dioica*, and *Anagalis arvensis* is a rich source of bioactive metabolites with antimycotic agents against large numbers of dermatophytes including *M. canis*, *M. gypseum*, *T. mentagrophytes*, *T. rubrum*, *T. schoenleinii*, and *E. floccosum* with MFCs ranged from 0.001 to 0.016 mg/mL. As a result, this study validates the use of plants in the treatment of fungal infections and gives rise to the prospect of discovering novel antifungal agents derived from plants. Due to all *Trichophyton* species showing significant sensitivity against all ethanolic extracts of all plants under study, *T. rubrum* showed a higher resistance towards the ethanolic extracts of *P. albicans*, *T. hirsuta*, *and U. maritima* at a concentration of 12.5 *μ*g/mL was selected for SEM studies. The deformation and fusion increased in the exposed hyphae with increased extract concentration. This altered morphology might be attributed to the natural bioactive compounds present in these extracts interfering with the architecture of the fungal morphology, cell membranes, and the functional groups on the surface of *T. rubrum*. Our results are in accordance with Zheng et al. [66], who showed that cis-trans citral isomers isolated from medicinal plants revealed antifungal activities against *T. rubrum* through higher cellular leakage rates, increased membrane damage, and morphological changes, presence of conidia with damaged membranes, and more distinct shriveled mycelium in SEM. Hence, S.E.M. analysis for studying and understanding of these changes in morphology and surface characteristics of fungus after treatment or stress may provide evidence of the efficacy of antifungal therapy [1]. Behbehani et al. [67] reported that after treatment with the plant extract, serious changes in the fungal morphology could be observed due to the approaches adopted by fungi to thrive well even in the presence of antifungal stress molecules in each extract by masking their toxic impact under stress conditions to shield themselves from the harsh environment. Antimycotic activity as MICs and MFCs of extracts was further confirmed through SEM's altered architecture of the dermatophytes under study. In accordance with our results, Taha et al. [5], by using scanning electron microscopy (SEM) analysis, revealed the morphological changes in hyphae of 30 clinical dermatophytes, including *Trichophyton* and *Microsporum* species exposed to ethanolic and acetone extracts of henna leaves and these structural changes including disintegrating, distortion, twisty, swollen, crushing of hyphae with waving and depressing on hyphal surfaces at 25 to 75 *μ*g/mL of ethanol or acetone extract.

Cancer is one of the leading causes of death globally, and the discovery of new anticancer drugs is the most important need in recent times. Natural products have been recognized as effective in fighting against various diseases, including cancer, for over 50 years [4]. Similar to our results, Nazarizadeh et al. [68] revealed that ethanol extract of *P. major* (1 *μ*g/mL) was cytotoxic against the ovary and cervix carcinoma stimulating the proliferation of nasopharynx carcinoma along with other activities like anti-inflammatory, wound healing, hematopoietic and antifatigue. Notably, Gálveza et al. [69] reported the strong cytotoxicities of the ethanolic extracts of different *Plantago* species against UACC-62 and MCF-7 cells with IC50 equal to 34 and 32 *μ*g/mL, respectively. Previously, various authors reported that *U. maritima* is well known for its applications in traditional medicine and is typical for the treatment of malignancies due to the selective killing of different malignant cells through various processes like apoptosis, antiproliferation, and cell cycle arrest instead of necrosis in different types of cancer cell lines including human leukemia, breast cancer cells (MCF-7, and MDA-MB2311), prostate, melanoma, pancreatic, lung, liver, glioblastoma, and colon in a dose-dependent mode [14, 70–72]. The high toxicity of some cancer chemotherapy drugs, in addition to their unfavorable side effects and drug resistance, drives up the demand for natural compounds as new anticancer drugs. Then these plants, *T. hirsuta*, *U. maritima*, and *P. albicans* appear to be among the preferred drugs in the future for treating these deadly diseases.

## 5. Conclusions

Dermatophyte infections are very common, and cancer is one of the primary causes of mortality globally. Hence, the discovery of new antimycotic and anticancer drugs is an essential need in recent times. Our study emphasizes the importance of identifying enzymatic and nonenzymatic virulence factors among clinical isolates of dermatophytes that contribute a major role to the emergence of antifungal drug resistance. This work provides an overview of the phytochemistry, pharmacological activities, and other potential applications of the aerial parts of ethnomedicinal plants, *T. hirsuta*, *U. maritima*, and *P. albicans*. Phytochemical screening showed that these plants are rich in polyphenols, tannins, flavonoids, alkaloids, carotenoids, luteinss, saponins, and polysaccharides and are known to have a wide range of biological activity. Furthermore, GC-MS analysis using the ethanolic extracts of these plants showed the presence of various bioactive compounds that correspondingly contribute to their exerting specific biological activities and potential therapeutic effects. The results of the current investigation clearly indicate the potential medicinal use of the ethanol extracts of *T. hirsuta*, *U. maritima*, and *P. albicans* (aerial parts) as antimycotic agents against a large number of dermatophytes and as antiproliferative agents against different types of cancer. Thus, this work confirms the importance of plants as a powerful source of diverse bioactive pharmaceutical metabolites that are of great importance for the development of new drugs and will be beneficial to investigators/scientists that are globally involved in the development of safe, effective, natural, and economical therapeutic agents/drugs against various fungal and cancerous diseases in the field of drug discovery.

## Figures and Tables

**Table 1:** Preliminary phytochemical analysis of the ethyl acetate, ethanol, and acetone extracts of *P. albicans*, *T. hirsuta*, and *U. maritima.*

Phytochemical composition (mg/g)	Plant								
	*P. albicans*			*T. hirsuta*			*U. maritima*		
	Ethyl acetate extract	Ethanol extract	Acetone extract	Ethyl acetate extract	Ethanol extract	Acetone extract	Ethyl acetate extract	Ethanol extract	Acetone extract
Polyphenols	40.31 ± 0.03	180.61 ± 0.08	162.58 ± 0.14	53.22 ± 0.03	250.09 ± 0.17	192.16 ± 0.11	71.16 ± 0.08	362.74 ± 0.51	329.58 ± 0.45
Flavonoids	39.28 ± 0.06	153.19 ± 0.35	124.92 ± 0.30	31.19 ± 0.01	141.83 ± 0.36	119.28 ± 0.25	34.75 ± 0.10	169.30 ± 0.48	112.21 ± 0.42
Tannins	3.51 ± 0.21	11.80 ± 0.69	9.84 ± 0.36	2.99 ± 0.17	9.00 ± 0.48	7.63 ± 0.38	4.18 ± 0.27	15.96 ± 0.76	11.96 ± 0.71
Carotenoids	0.66 ± 0.14	3.12 ± 0.38	2.46 ± 0.32	0.62 ± 0.19	5.51 ± 0.60	5.02 ± 0.66	1.77 ± 0.25	6.95 ± 0.83	5.78 ± 0.75
Lutein	0.31 ± 0.0	0.85 ± 0.16	0.76 ± 0.11	0.19 ± 0.03	0.49 ± 0.22	0.34 ± 0.17	0.25 ± 0.06	1.12 ± 0.47	0.90 ± 0.34
Saponins	1.42 ± 0.77	4.95 ± 2.15	4.78 ± 2.09	1.73 ± 0.64	5.23 ± 2.41	3.95 ± 2.25	2.16 ± 1.01	6.89 ± 3.16	6.05 ± 3.07
Alkaloids	0.65 ± 0.62	2.78 ± 1.90	2.36 ± 1.70	0.47 ± 0.50	2.76 ± 2.13	2.65 ± 2.19	1.10 ± 0.82	5.64 ± 4.08	5.31 ± 4.16

**Table 2:** Phytochemical analysis of *P. albicans* L. by GC-Mass analysis.

No.	Constituent	RT (min)	Area %
1	Glycerin	4.892	2.15
2	Naphthalene	7.936	6.12
3	4-Hydroxy-3-methoxybenzoic acid	9.350	1.90
4	Dodecanoic acid, methyl ester (lauric acid)	10.898	6.26
5	Diathiapentene	11.982	1.10
6	9-Eicosene, (E)-	12.120	1.17
7	Cinnamic acid	12.514	12.16
8	Eicosanoic acid, methyl ester (arachidic acid)	14.871	2.26
9	Octadecanoic acid, methyl ester	15.862	1.24
10	Phytol	16.142	10.21
11	Catechin	17.299	8.30
12	Benzofuranone	19.721	7.10
13	D-Carvone	21.364	2.13
14	Tetradecane	22.416	11.18
15	Trans-caryophyllene	26.981	13.20
16	Chlorogenic acid	29.142	6.30
17	Hexadecanoic acid	29.453	1.30
18	Hydroxy-B ionone	30.984	1.88
19	Pentadecanone	31.266	1.15
20	Gallic acid	31.544	1.24
21	Tetracosanoic acid	31.585	1.52

**Table 3:** Phytochemical analysis of *T. hirsuta* L. by GC-Mass analysis.

Number	Constituent	RT (min)	Area %
1	3,5-Dithiahexanol 5,5-dioxide	7.581	7.23
2	1-Octen-3-ol	8.426	0.50
3	1-Hexadecanol	9.942	2.00
4	Phenylethyl alcohol	10.133	0.47
5	Camphor	11.37	0.93
6	Lavandulyl acetate	12.91	4.43
7	Citronellyl formate	13.580	1.19
8	*β*-Bourbonene	14.930	2.12
9	Tetratetracontane	15.116	1.48
10	*β*-Cyclocitral	16.623	0.70
11	*α*-Pinene	16.678	0.61
12	Nonadecene	16.896	12.17
13	Germacrene-D	18.415	1.23
14	Thymohydroquinone dimethyl ether	18.731	2.11
15	*α*-Humulene	19.128	15.25
16	*α*-Guaiene	21.050	1.34
17	Alloaromadendrene	21.243	13.60
18	*σ*-Cadinene	23.029	4.71
19	*α*-Gurjunene	24.114	1.10
20	Valencene	25.220	18.0
21	*β*-Selinene	27.392	2.15
22	*γ*-Gurjunene	28.604	1.65
23	*α*-Cedrene	29.380	3.30
24	Phenol, 2,4-bis (1,1-dimethylethyl)-	31.416	1.14
25	Tetradecamethylheptasiloxane	32.082	0.59

**Table 4:** Phytochemical analysis of *U. maritima* L. by GC-Mass analysis.

No.	Constituent	RT (min)	Area %
1	Cyclohexasiloxane, dodecamethyl	9.116	0.76
2	2-Pentyl-furan	9.424	0.58
3	3-Carene	10.994	0.63
4	Phenylalcohol	11.5	0.35
5	Tetradecanoic acid, methyl ester	11.3	0.91
6	Borneol	12.7	1.28
7	Torreyol	12.502	4.11
8	*α*-Cadinol	12.241	7.43
9	Terpinolene	13.1	0.68
10	Linalyl acetate	13.5	1.00
11	Carveol	13.721	9.74
12	2-Hydroxy-4-methoxybenzaldehyde	15.081	0.42
13	Gamma-Sitosterol	14.9345	0.74
14	Geraniol	15.4	2.20
15	*α*–Pinene	16.745	0.96
16	5-Isopropyl-2-methyl phenol	16.7	15.21
17	1,2-Benzendicarboxylic acid, diisooctyl ester	16.7	6.22
18	Tetrafluorophthalonitrile	17.0	7.62
19	p-Vinylguaiacol	18.1	0.83
20	Eugenol	19.437	13.25
21	*β*-Myrcene	19.1	0.70
22	Retinol	22.3	0.30
23	Espatulenol	23.5	0.45
24	Hexadecanoic acid	39.231	7.60
25	Cyclohexane, 1,4-dimethyl-2-octadecyl-	38.37	3.29
26	*β*-Sesquiphellandrene	44.33	0.43
27	Oleic acid	36.65	0.72
28	*β*-Elemene	40.55	6.58
29	*β*-Caryophyllene	41.77	4.29
30	Octadecane, 3-ethyl-5-(2-ethylbutyl)-	32.549	0.51

**Table 5:** Specificity and efficiency of various culture media for isolation of dermatophytes.

Clinical fungal isolates	Growth			Color change		
	RSM	DTM	DIM	RSM	DTM	DIM
Total dermatophytic fungi	253	253	253	208	220	253
*T. rubrum*	41	41	41	29	33	41
*T. mentagrophytes*	40	40	40	31	36	40
*T. tonsurance*	34	34	34	29	29	34
*T. soudanense*	13	13	13	13	11	13
*T. vaoundei*	6	6	6	6	6	6
*T. erinacei*	5	5	5	3	4	5
*M. audouinii*	9	9	9	7	8	9
*M. gypseum*	14	14	14	14	11	14
*M. ferrogenium*	11	11	11	10	11	11
*M. cookie*	19	19	19	16	13	19
*M. canis*	16	16	16	13	16	16
*M. persicolor*	14	14	14	10	12	14
*M. gallinae*	7	7	7	7	7	7
*M. racemosum*	8	8	8	7	8	8
*E. floccosum*	16	16	16	13	15	16
Total nondermatophytic fungi	30	18	3	15	17	1
Total *Candida* sp.	16	10	3	11	9	1
Spore forming bacilli	5	4	0	1	4	0
*Staphylococcus aureus*	4	2	0	2	2	0
*Escherichia coli*	5	2	0	1	2	0

**Table 6:** Virulence factors formation in dermatophytic fungi on different human substrates.

Dermatophytic fungi	Virulence factors production														
	Stratum corneum					Human hair					Human nail				
	K	L	P	H (A_550_)	M	K	L	P	H (A_550_)	M	K	L	P	H (A_550_)	M
*T. rubrum*	20.35	22.46	29.91	0.69	0.63	18.13	22.56	28.01	0.69	0.60	16.00	23.46	29.55	0.68	0.77
*T. mentagrophytes*	11.82	54.56	15.67	0.68	0.21	10.06	53.00	15.00	0.68	0.42	8.39	48.55	14.07	0.68	0.45
*T. tonsurance*	19.26	39.12	27.59	0.39	0.68	13.64	40.13	27.45	0.39	0.58	13.91	40.16	27.01	0.38	0.71
*T. soudanense*	13.25	29.45	16.19	0.46	0.15	12.63	25.49	18.16	0.46	0.49	9.49	26.37	11.44	0.45	0.44
*T. vaoundei*	20.14	36.19	24.14	0.49	0.39	17.41	29.60	21.58	0.47	0.51	15.88	34.27	18.02	0.48	0.56
*T. erinacei*	14.57	57.38	19.33	0.42	0.55	14.02	50.01	16.41	0.42	0.50	14.00	51.27	17.40	0.41	0.69
*M. audouinii*	17.09	31.26	21.02	0.53	0.32	15.00	31.16	19.30	0.52	0.46	15.61	32.01	19.31	0.52	0.59
*M. gypseum*	18.94	33.16	19.89	0.60	0.57	16.84	30.19	20.34	0.59	0.56	15.68	36.02	19.70	0.59	0.68
*M. ferrogenium*	19.29	60.12	28.64	0.49	0.38	15.65	55.11	25.31	0.49	0.40	14.96	60.00	28.12	0.48	0.50
*M. cookie*	23.56	36.19	26.01	0.43	0.29	21.63	42.88	25.01	0.42	0.35	22.10	38.61	25.01	0.42	0.56
*M. canis*	19.87	19.09	26.91	0.39	0.23	15.01	19.65	25.09	0.39	0.40	13.98	23.47	25.41	0.38	0.67
*M. persicolor*	14.99	62.25	12.90	0.50	0.33	10.71	60.72	17.50	0.49	0.39	9.01	54.79	17.67	0.49	0.57
*M. gallinae*	14.36	15.24	18.61	0.43	0.46	10.58	16.49	15.15	0.42	0.41	9.18	10.91	15.99	0.42	0.76
*M. racemosum*	9.28	60.34	14.10	0.42	0.19	5.86	56.02	10.06	0.40	0.30	7.95	60.18	13.09	0.39	0.47
*E. floccosum*	22.08	40.65	25.74	0.62	0.62	19.08	53.84	25.00	0.61	0.60	17.90	51.23	25.40	0.62	0.74

**Table 7:** Antifungal activity of different plants extracts at different concentrations against dermatophytic fungi.

Plant extract (*μ*g/mL)	Dermatophytic fungal toxicity (%)														
	*T. rubrum*	*T. tonsurance*	*T. mentagrophytes*	*T. soudanense*	*T. vaoundei*	*T. erinacei*	*M. audouinii*	*M. gypseum*	*M. ferrogenium*	*M. cookie*	*M. canis*	*M. persicolor*	*M. gallinae*	*M. racemosum*	*E. floccosum*
*P. albicans*															
EtOAc ex.															
12.5	19.53 ± 0.12	13.40 ± 0.19	10.34 ± 0.08	21.16 ± 0.26	20.40 ± 0.24	10.32 ± 0.10	17.00 ± 0.15	9.17 ± 0.06	12.28 ± 0.11	20.00 ± 015	15.0 ± 0.06	18.0 ± 0.11	13.0 ± 0.09	23.18 ± 0.18	14.20 ± 0.18
25	36.11 ± 0.36	23.10 ± 0.31	29.26 ± 0.40	30.24 ± 0.38	36.16 ± 0.45	29.18 ± 0.24	40.29 ± 0.36	16.58 ± 0.13	19.15 ± 0.18	27.92 ± 0.18	27.4 ± 0.15	21.9 ± 0.19	29.0 ± 0.18	30.08 ± 0.23	16.94 ± 0.19
50	48.62 ± 0.47	37.51 ± 0.49	45.3 ± 0.51	50.80 ± 0.56	57.51 ± 0.62	45.63 ± 0.50	50.81 ± 0.53	27.90 ± 0.20	24.23 ± 0.23	31.16 ± 0.30	40.0 ± 0.38	33.8 ± 0.27	45.0 ± 0.32	36.14 ± 0.27	28.15 ± 0.23
Ethanol ex.															
12.5	36.31 ± 0.25	41.29 ± 0.19	66.16 ± 0.42	80.7 ± 0.61	70.53 ± 0.56	86.7 ± 0.54	75.0 ± 0.60	96.7 ± 0.60	63.32 ± 0.42	90.0 ± 0.75	80.25 ± 0.61	66.1 ± 0.41	90.29 ± 0.65	68.22 ± 0.45	19.42 ± 0.11
25	70.00 ± 0.49	76.72 ± 0.51	93.50 ± 0.60	100.0 ± 0.50	93.0 ± 0.78	100.0 ± 0.70	100.0 ± 0.64	100.0 ± 0.58	100.0 ± 0.50	100.0 ± 0.80	100.0 ± 0.73	100.0 ± 0.50	100.0 ± 0.60	100.0 ± 0.50	30.16 ± 0.23
50	85.0 ± 0.65	100.0 ± 0.70	100.0 ± 0.60	100.0 ± 0.48	100.0 ± 0.83	100.0 ± 0.78	100.0 ± 0.57	100.0 ± 0.63	100.0 ± 0.50	100.0 ± 0.69	100.0 ± 0.68	100.0 ± 0.53	100.0 ± 0.60	100.0 ± 0.51	32.92 ± 0.27
Acetone ex.															
12.5	40.10 ± 0.26	14.0 ± 0.05	45.18 ± 0.17	76.0 ± 0.42	52.1 ± 0.22	60.0 ± 0.23	56.28 ± 0.59	71.0 ± 0.48	64.16 ± 0.41	56.31 ± 0.47	80.22 ± 0.51	63.50 ± 0.38	65.19 ± 0.69	70.0 ± 0.42	6.15 ± 0.06
25	56.27 ± 0.31	30.3 ± 0.13	70.3 ± 0.39	90.0 ± 0.51	91.0 ± 0.35	77.0 ± 0.30	77.90 ± 0.63	90.0 ± 0.50	80.37 ± 0.50	80.10 ± 0.54	89.53 ± 0.46	75.63 ± 0.44	90.0 ± 0.60	83.50 ± 0.48	18.23 ± 0.22
50	80.0 ± 0.47	65.1 ± 0.40	89.0 ± 0.45	100.0 ± 0.56	100.0 ± 0.39	85.0 ± 0.37	90.00 ± 0.60	100.0 ± 0.53	100.0 ± 0.59	93.24 ± 0.60	100.0 ± 0.55	90.0 ± 0.51	100.0 ± 0.73	100.0 ± 0.52	30.42 ± 0.27
*T. hirsuta*															
EtOAc ex.															
12.5	22.14 ± 0.11	14.16 ± 0.09	11.72 ± 0.06	19.00 ± 0.14	15.38 ± 0.12	26.14 ± 0.18	0.0 ± 0.0	0.0 ± 0.0	0.0 ± 0.0	6.92 ± 0.05	11.00 ± 0.05	2.61 ± 0.0	3.15 ± 0.04	0.0 ± 0.0	23.26 ± 0.27
25	29.23 ± 0.16	40.23 ± 0.18	24.96 ± 0.12	33.16 ± 0.22	22.40 ± 0.17	35.23 ± 0.26	0.0 ± 0.0	7.16 ± 0.04	9.16 ± 0.08	15.40 ± 0.10	25.71 ± 0.19	4.75 ± 0.0	6.90 ± 0.09	0.0 ± 0.0	40.42 ± 0.33
50	48.70 ± 0.24	51.02 ± 0.25	43.10 ± 0.20	50.10 ± 0.31	37.66 ± 0.23	59.67 ± 0.34	12.60 ± 0.09	12.54 ± 0.10	20.34 ± 0.21	23.71 ± 0.18	30.92 ± 0.23	10.83 ± 0.07	14.55 ± 0.17	7.42 ± 0.03	61.58 ± 0.39
Ethanol ex.															
12.5	50.69 ± 0.20	80.42 ± 0.34	60.10 ± 0.55	90.16 ± 0.31	61.0 ± 0.43	70.21 ± 0.45	18.51 ± 0.14	45.19 ± 0.21	13.60 ± 0.19	20.26 ± 0.24	41.19 ± 0.30	24.62 ± 0.15	16.43 ± 0.23	10.0 ± 0.01	85.0 ± 0.26
25	100.0 ± 0.0	100.0 ± 0.40	100.0 ± 0.61	100.0 ± 0.30	100.0 ± 0.67	100.0 ± 0.52	25.34 ± 0.20	61.44 ± 0.30	22.62 ± 0.28	33.0 ± 0.40	53.42 ± 0.41	30.38 ± 0.22	28.50 ± 0.34	15.0 ± 0.06	100.0 ± 0.35
50	100.0 ± 0.0	100.0 ± 0.43	100.0 ± 0.60	100.0 ± 0.34	100.0 ± 0.70	100.0 ± 0.50	36.67 ± 0.34	69.20 ± 0.37	32.15 ± 0.36	47.0 ± 0.46	75.11 ± 0.53	40.79 ± 0.37	39.62 ± 0.41	31.3 ± 0.14	100.0 ± 0.32
Acetone ex.															
12.5	45.40 ± 0.22	32.18 ± 0.27	43.50 ± 0.34	47.09 ± 0.25	58.74 ± 0.40	59.3 ± 0.39	10.10 ± 0.04	30.72 ± 0.16	10.28 ± 0.08	15.45 ± 0.15	30.02 ± 0.20	19.00 ± 0.12	16.15 ± 0.09	5.59 ± 0.16	57.16 ± 0.63
25	78.23 ± 0.39	60.51 ± 0.32	90.21 ± 0.40	75.18 ± 0.36	92.0 ± 0.50	85.0 ± 0.57	15.34 ± 0.09	58.23 ± 0.29	17.50 ± 0.16	26.21 ± 0.26	45.24 ± 0.26	25.16 ± 0.19	25.42 ± 0.21	17.84 ± 0.30	79.74 ± 0.71
50	100.0 ± 0.46	100.0 ± 0.40	100.0 ± 0.46	100.0 ± 0.50	100.0 ± 0.61	100.0 ± 0.52	28.62 ± 0.18	60.10 ± 0.40	23.26 ± 0.24	40.30 ± 0.32	60.79 ± 0.38	37.51 ± 0.24	35.19 ± 0.29	26.00 ± 0.35	100.0 ± 0.79
*U. maritima*															
EtOAc ex.															
12.5	22.37 ± 0.12	19.12 ± 0.08	30.73 ± 0.29	19.0 ± 0.16	22.74 ± 0.20	30.11 ± 0.0	27.63 ± 0.16	26.17 ± 0.18	19.50 ± 0.17	31.00 ± 0.26	18.1 ± 0.06	13.25 ± 0.10	19.7 ± 0.17	21.66 ± 0.20	43.47 ± 0.22
25	56.23 ± 0.38	35.92 ± 0.26	42.20 ± 0.34	40.0 ± 0.29	50.50 ± 0.32	55.60 ± 0.1	38.19 ± 0.21	48.40 ± 0.27	30.82 ± 0.26	50.08 ± 0.37	42.0 ± 0.23	25.60 ± 0.18	30.0 ± .24	35.21 ± 0.29	71.51 ± 0.45
50	69.40 ± 0.42	54.31 ± 0.38	56.81 ± 0.51	69.0 ± 0.33	61.19 ± 0.40	68.00 ± 0.9	50.00 ± 0.29	65.00 ± 0.43	49.63 ± 0.32	62.46 ± 0.42	50.0 ± 0.29	46.39 ± 0.28	61.0 ± 0.42	50.74 ± 0.34	80.20 ± 0.51
Ethanol ex.															
12.5	61.0 ± 0.0	100.0 ± 0.0	75.0 ± 0.05	100.0 ± 0.0	100.0 ± 0.0	100.0 ± 0.0	100.0 ± 0.0	100.0 ± 0.0	100.0 ± 0.4	100.0 ± 0.0	100.0 ± 0.0	100.0 ± 0.0	100.0 ± 0.0	100.0 ± 0.0	100.0 ± 0.0
25	100.0 ± 0.0	100.0 ± 0.0	100.0 ± 0.0	100.0 ± 0.0	100.0 ± 0.0	100.0 ± 0.0	100.0 ± 0.0	100.0 ± 0.0	100.0 ± 0.0	100.0 ± 0.0	100.0 ± 0.0	100.0 ± 0.0	100.0 ± 0.0	100.0 ± 0.0	100.0 ± 0.0
50	100.0 ± 0.0	100.0 ± 0.0	100.0 ± 0.0	100.0 ± 0.0	100.0 ± 0.0	100.0 ± 0.0	100.0 ± 0.0	100.0 ± 0.0	100.0 ± 0.0	100.0 ± 0.0	100.0 ± 0.0	100.0 ± 0.0	100.0 ± 0.0	100.0 ± 0.0	100.0 ± 0.0
Acetone ex.															
12.5	60.0 ± 0.0	100.0 ± 0.0	70.0 ± 0.0	100.0 ± 0.0	100.0 ± 0.0	100.0 ± 0.0	100.0 ± 0.0	100.0 ± 0.0	100.0 ± 0.0	100.0 ± 0.0	85.0 ± 0.0	100.0 ± 0.0	100.0 ± 0.0	100.0 ± 0.0	100.0 ± 0.0
25	100.0 ± 0.0	100.0 ± 0.0	100.0 ± 0.0	100.0 ± 0.0	100.0 ± 0.0	100.0 ± 0.0	100.0 ± 0.0	100.0 ± 0.0	100.0 ± 0.0	100.0 ± 0.0	100.0 ± 0.0	100.0 ± 0.0	100.0 ± 0.0	100.0 ± 0.0	100.0 ± 0.0
50	100.0 ± 0.0	100.0 ± 0.0	100.0 ± 0.0	100.0 ± 0.0	100.0 ± 0.0	100.0 ± 0.0	100.0 ± 0.0	100.0 ± 0.0	100.0 ± 0.0	100.0 ± 0.0	100.0 ± 0.0	100.0 ± 0.0	100.0 ± 0.0	100.0 ± 0.0	100.0 ± 0.0
DMSO (1%)	0.0 ± 0.0	0.0 ± 0.0	0.0 ± 0.0	0.0 ± 0.0	0.0 ± 0.0	0.0 ± 0.0	0.0 ± 0.0	0.0 ± 0.0	0.0 ± 0.0	0.0 ± 0.0	0.0 ± 0.0	0.0 ± 0.0	0.0 ± 0.0	0.0 ± 0.0	0.0 ± 0.0
Clotrimazole 200 *μ*g/mL	100.0 ± 0.0	100.0 ± 0.0	100.0 ± 0.0	100.0 ± 0.0	100.0 ± 0.0	100.0 ± 0.0	100.0 ± 0.0	100.0 ± 0.0	100.0 ± 0.0	100.0 ± 0.0	100.0 ± 0.0	100.0 ± 0.0	100.0 ± 0.0	100.0 ± 0.0	100.0 ± 0.0

## Data Availability

All data generated or analyzed during this investigation are included in this research article.
